# The Associations between Sleep Duration, Academic Pressure, and Depressive Symptoms among Chinese Adolescents: Results from China Family Panel Studies

**DOI:** 10.3390/ijerph18116134

**Published:** 2021-06-06

**Authors:** Tong Zhou, Gang Cheng, Xihong Wu, Rui Li, Chao Li, Gang Tian, Simin He, Yan Yan

**Affiliations:** Department of Epidemiology and Medical Statistics, Xiangya School of Public Health, Central South University, Xiangya Road 110, Changsha 410078, China; tongzhou@csu.edu.cn (T.Z.); gangcheng@csu.edu.cn (G.C.); wuxihong@csu.edu.cn (X.W.); 206912061@csu.edu.cn (R.L.); Jenniferchaoli@csu.edu.cn (C.L.); tiangang@csu.edu.cn (G.T.); hesimin1969@csu.edu.cn (S.H.)

**Keywords:** depressive symptom, sleep duration, academic pressure, adolescents

## Abstract

Depressive symptoms are a common mental health problem among adolescents, which may affect their physical and mental health development and impose heavy burdens on individual families and society. This study aimed to examine the associations between sleep duration, academic pressure, and depressive symptoms among Chinese adolescents and to construct the mediation model to explore the mediating effect of sleep duration. The data are from the China Family Panel Studies (CFPS). Methodologically, the aforementioned associations were explored by constructing a structural equation model and applying multivariate multilevel logistic regression. In this study, we found that approximately 6.49% of the 3724 Chinese adolescents had depressive symptoms. Sleep duration of <6 h/night (OR = 2.39, 95%CI = 1.33–4.32) and high/maximum academic pressure (high: OR = 1.43, 95%CI = 1.02–1.99; maximum: OR = 2.43, 95%CI = 1.58–3.73) were both associated with an increased risk of depressive symptoms in adolescents. Meanwhile, the multiplicative interaction between sleep duration and academic pressure was significantly associated with depressive symptoms in adolescents (*p* < 0.001). The sleep duration played a partial mediating role in the relationship between academic pressure and depressive symptoms (a*b = 0.006, 95%BootCI = 0.001–0.012). Our study highlights that it is essential to mitigate the academic pressure of adolescents to increase their sleep duration and further reduce the occurrence of depressive symptoms by adopting corresponding preventive measures.

## 1. Introduction

Adolescence is a transitional period from childhood to adulthood that is susceptible to mental and physical health problems [1]. It is recognized as a period involving a significant risk for the onset of depression [2]. Depressive symptoms are a common mental health problem among adolescents all over the world. About 2.6% of children and adolescents worldwide suffer from depression [3]. In China, the prevalence of depressive symptoms in children and adolescents ranges from 4% to 41%, and the pooled prevalence of depressive symptoms is 19.85% [4]. The World Health Organization (WHO) expects that the disease burden of depression will top all other diseases by 2030 [5]. Depressive symptoms in adolescence have a long-term impact on cognitive ability development. It may increase the risk of depression and suicide, placing a heavy burden on individual families and society [6]. Therefore, there is a vital need to detect and prevent this disease.

Sleep duration, as an important indicator of health [7], is essential for maintaining an optimal health condition in children and adolescents. All over the world today, sleep duration and sleep quality have become a significant public health problem for adolescents. The daily duration of sleep recommended by the American Academy of Sleep Medicine (AASM) for adolescents (13–18 years) is 8–10 h/night [8]; consistently, the National Sleep Foundation (NSF) also mentions a sleep duration of 8–10 h/night for teenagers (14–17 years). Nevertheless, because of psychological factors such as stress and depression, many teenagers are significantly short of sleep, compared to the recommended level [9]. Poor sleep is detrimental to adolescents’ health and can lead to obesity, headaches, cardiovascular diseases, depression, and fatigue [10]. A study conducted on Chinese adolescents revealed a U-shaped relationship between sleep duration and depressive symptoms [11,12]. A survey of teenagers in the United States showed that insufficient sleep was associated with a number of health-risk behaviors, such as the current use of cigarettes, marijuana, feeling of sadness or hopelessness, and physical fight [13]. Thus, sufficient sleep is essential for preventing depression and health-risk behaviors in adolescents. Previous studies have shown that short sleep duration can affect mental health by disrupting the dorsal medial prefrontal cortex (DMPFC), which plays a vital role in emotion control [14].

It is well known that Chinese adolescents are under tremendous pressure of study and may even have to sacrifice their sleep time [15,16]. Some studies have found that teenagers do not sleep enough in general because of academic stress [17]. An international survey has demonstrated a positive correlation between the students’ academic pressure and mental health problems [16,18]. According to a study in Singapore, teenagers with high academic stress had more severe depression and anxiety symptoms after adjusting sleep duration [19]. In addition, sleep duration significantly moderates the relationship between school stress and adolescent psychological symptoms in adolescents, according to a 12-nation research project [18]. However, the relationship between academic pressure, sleep duration, and depressive symptoms among Chinese adolescents was rarely investigated. Therefore, we intended to use this large-scale nationwide study to estimate the associations between habitual sleep duration, academic pressure, and depressive symptoms among Chinese adolescents and to construct the mediation model to explore the mediating effect of sleep duration. Given the prevalence and burden of depressive symptoms during adolescence, there is a great significance to mitigate the potential risk factors of depression for adolescents.

As aforementioned, it is essential to assess the current situations and the underlying associations between sleep duration, academic stress, and depressive symptoms among Chinese adolescents. This study aimed to test the following hypotheses: (1) shorter sleep duration is related to more severe depressive symptoms; (2) excessive academic pressure is related to more severe depressive symptoms; (3) the association between academic pressure and depressive symptoms is mediated by sleep duration.

## 2. Materials and Methods

### 2.1. Study Design and Sample

The data were retrieved from the China Family Panel Studies (CFPS), which were funded by the 985 Program of Peking University and carried out by the Institute of Social Science Survey (ISSS) of Peking University [20]. The CFPS is a nationally representative, the annual longitudinal project reviewed and approved by the ISSS of Peking University. All participants were requested to provide written informed consent before participating in the research. The CFPS surveyed approximately 15,000 households nationwide, using a multistage probability proportional-to-size sampling method, and interviewed all family members in each sampled household. The survey questionnaire was designed to gather individual, family, and community-level information on demographic and socioeconomic variables and information on the respondents’ health conditions [21]. Only anonymized data were released to researchers. The included data covered 37,147 Chinese individuals residing in 621 villages/communities from 25 of China’s 30 provinces, representing 95% of the total population in China. The CFPS mainly adopted face-to-face interviews aided by the computer-assisted personal interviewing (CAPI) technology. 

During all stages of data collection, the research team attempted to ensure data quality through telephone checks, field checks, audio recording checks, interview reviews, and interim statistical analyses. As aforementioned, the 2018 CFPS surveyed about 15,000 households and collected nearly 44,000 copies of questionnaires, of which 22% were collected by phone interviews, and the rest were collected by face-to-face interviews. Based on the national follow-up survey of households from 2010 to 2016, in 2018, the cross-sectional response rate was 67.4%, the cross-round follow-up rate was 80.8%, and the completion rate in 2018 was 64.5%. The WHO defines adolescents as individuals aged 10–19 years. According to the questionnaire of CFPS and Chinese adolescent research, we selected the participants aged 11–19 years from the respondents who completed the full 8-question version of the Center for Epidemiologic Studies Depression (CES-D) questionnaire in 2018. After eliminating independent variables containing missing values, our final analytical sample consisted of 3724 respondents in total. 

The project was retrieved from the CFPS website for public access to secondary data (https://www.isss.pku.edu.cn/cfps/index.htm, accessed on 5 June 2021), which excludes all identifiable information about individual participants. All participants were asked to provide written informed consent before completing the survey.

### 2.2. Variables and Definitions

#### 2.2.1. Measurement of Depressive Symptoms

In the 2018 CFPS, the depression symptoms were measured using the Center for Epidemiology Scale for Depression (CES-D) [22]. Specifically, CES-D 8 (including two positive and six negative questions) was used [23], which contained four subscales: somatic symptoms, interpersonal relations, depressed affect, and positive affect. The respondents were asked to rate how they experienced the specified emotions or behaviors in the past week: (1) I felt depressed; (2) I felt that everything I did was an effort; (3) My sleep was restless; (4) I was happy; (5) I felt lonely; (6) I enjoyed life; (7) I felt sad; (8) I could not get “going.” The rating varied from 0 to 3 for each question (0 = never; 1 = sometimes, 1–2 days; 2 = often, 3–4 days; 3 = most of the time, 5–7 days). Responses to negative emotions were assigned 0, 1, 2, and 3, and responses to the two positive emotions were assigned 3, 2, 1, and 0. All the scores were aggregated on a scale of 0 to 24. Depression is a persistent phenomenon, and a higher score indicates a higher level of depressive symptoms. In this study, the score of 9 was set to be the cutoff point for clinically significant depressive symptoms [24]. At present, the CES-D is deemed a practical/reliable depression screening tool for the Chinese population, which has been validated and widely used by previous studies targeting Chinese adolescents [25,26]. The depressive symptoms in this study were diagnosed as one week after a depressive event according to the CES-D criteria rather than a diagnosis of depression. The Cronbach’s alpha of this study was 0.712, indicating good reliability.

#### 2.2.2. Measurement of Sleep Duration 

Sleep duration was assessed by the question: “How many hours did the participants sleep during school days and weekends, respectively?” The sleep duration was calculated by (hours on school days * 5 + hours on weekends * 2)/7 [27] and was categorized as <6 h (short sleep duration), 6–8 h (average sleep duration), and >8 h (long sleep duration). The responses of 6–8 h were marked as standard rather than the recommended sleep duration, and this option was taken as the reference group. Self-reported TST is an effective measure of TST for adolescents and is correlated with more objective criteria (such as actigraphy). There was no significant difference between the two measurement results, and the Pearson correlation coefficient was (0.53–0.77) [28].

#### 2.2.3. Measurement of Academic Pressure

Academic pressure was assessed based on the adolescents’ subjective perceptions of study stress at school, and the responses were coded as follows: 1 = minimum; 2 = low; 3 = moderate; 4 = high; 5 = maximum.

#### 2.2.4. Measurement of Covariates

According to previous research, potential confounding variables including age, gender, household socioeconomic status, exercise duration, internet use time (for entertainment), midday napping, class rank [29], interpersonal relationship, smoking [13], drinking [30], and BMI [31] were selected. The household socioeconomic status was classified by the percentile of family per capita income as follows: 1 = low; 2 = moderate; 3 = high. The exercise duration was assessed by the question: “How much time in total do you exercise each week?” The responses were converted into minutes of exercise per day and classified as follows: <30 min; 30–60 min; >60 min. The internet use time (for entertainment) was assessed by the question: “How many hours per day do you spend on the Internet in your spare time?” The smoking and drinking status was assessed by the question: “Have you used at least one cigarette/drink during the recent month?” The responses were coded as follows: 1 = yes; 2 = no. The interpersonal relationship was classified as follows: 1 = poor, 2 = average, 3 = good. The class rank was assessed by the question: “How did you rank in your class on the last comprehensive examination?” The responses were classified as follows: 0–10%; 11–25%; 26–50%; 51–75%; 76–100%. The self-reported height and weight were calculated into BMI z-scores to determine the participant’s weight status. The age-gender-adjusted BMI standard deviation scores (BMI z-scores) were computed and distributed by referring to the WHO growth charts and the official WHO z-score calculator. Based on the WHO recommendation, overweight was defined as a BMI z-score > 1; obesity was defined as a BMI z-score > 2; normal weight was defined as a BMI z-score ≤ 1 or ≥ −2; underweight was defined as a BMI z-score < −2.

### 2.3. Statistical Analysis

**Figure 1:**
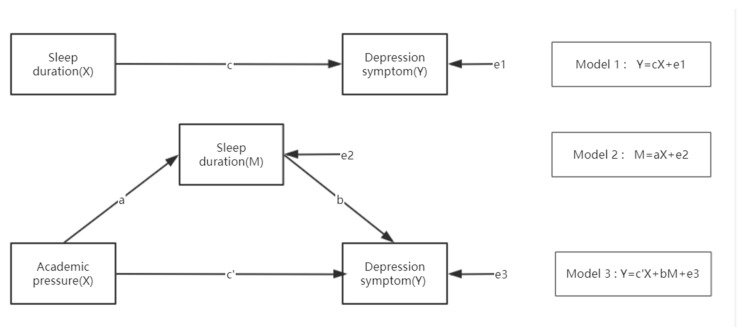
The framework of the potential mediating effect of sleep duration on the association between academic pressure and depressive symptoms.

First, descriptive analyses were used to describe baseline characteristics. The mean and standard deviations for continuous variables and percentages for categorical variables were used to describe sample characteristics. The t-test or one-way analysis of variance (ANOVA) was used for CES-D scores, and the chi-square test was used for depressive symptoms. Second, the variables were analyzed using the univariable multilevel logistic regression model; variables that were significant at the 0.10 level in univariable analyses were included in the multivariable multilevel logistic regression model. Third, the interaction term multiplied by sleep duration and academic pressure was included in the multivariable multilevel logistic regression model for testing and *p*-value calculation. Fourth, if the multiplicative interaction term was significantly associated with depressive symptoms, a further stratification analysis would be performed to evaluate the relationship between sleep duration and depressive symptoms at different levels of academic pressure. Fifth, AMOS 26.0 was used to build the structural equation model to explore the mediating effect of sleep duration on the relationship between academic pressure and depressive symptoms. As shown in Figure 1, the mediating effect model consists of three types of regression models. Specifically, the structural equation model was adjusted for age, gender, exercise duration, internet use time (for entertainment), class rank, smoking, drinking, and popularity relations. Model 1 is the regression model of academic pressure (X) and depressive symptoms (Y). Model 2 is the regression model of academic pressure (X) and sleep duration (M). Model 3 is the regression model of academic pressure X and sleep duration M and depressive symptoms Y (Figure 1).

The multiple imputation method was used to estimate the missing values. All statistical tests were two-sided, and *p* < 0.05 was considered statistically significant. Statistical analyses were performed using SPSS 26.0 (SPSS, Inc., Chicago, IL, USA). This study examined the hypothetical model using AMOS 26.0 (SPSS, Inc., Chicago, IL, USA) for the purpose of testing the structural equation model. 

## 3. Results

### 3.1. Descriptive Statistics

A total of 3724 adolescents were enrolled in this study. The mean CES-D8 score was 4.27 (SD: 3.11), and 242 (6.49%) subjects had depressive symptoms. The mean age of the total adolescents was 14.9 (SD: 2.6) years old. The proportions of adolescents who reported sleep duration of <6 h/night (*p* < 0.001), smoking (*p* < 0.001), drinking (*p* < 0.001), class rank (*p* < 0.001), academic pressure (*p* < 0.001), and popularity relation (*p* < 0.001) were statistically significant in CES-D8 scores. Adolescents who reported short sleep duration, long internet use time, low class rank, poor popularity relation, smoking, and drinking were more likely to have depressive symptoms (*p* < 0.001) (Table 1).

### 3.2. Sleep Duration, Academic Pressure, and Depressive Symptoms

Without adjusting for other variables, adolescents who reported a sleep duration of <6 h/night were at high risk of depressive symptoms (OR = 2.35, 95%CI = 1.32–4.17). After adjusting for age, gender, exercise duration, internet use time (for entertainment), class rank, smoking, drinking, and popularity relation, those who reported a sleep duration of <6 h/night will still at a high risk of depressive symptoms (OR = 2.39, 95%CI = 1.33–4.32). Moreover, adolescents who reported high and maximum academic pressure were more likely to be at a higher risk of depressive symptoms (high: OR = 1.43, 95%CI = 1.02–1.99; maximum: OR = 2.43, 95%CI = 1.58–3.73). After adjusting for the same factors, those who reported high and maximum academic pressure were still at a higher risk of depressive symptoms (*p* < 0.05), and the relationship between sleep duration(<6 h) and depressive symptoms remained statistically significant (*p* < 0.05) (Table 2).

Meanwhile, the multiplicative interaction between sleep duration and academic pressure had a statistically significant effect on depressive symptoms in both adjusted and unadjusted models (*p* < 0.001). We then carried out a stratified analysis of academic pressure. In the adjusted model, adolescents who reported moderate and maximum academic pressure with a sleep duration of <6 h/night had a significant risk of developing depressive symptoms (moderate: OR = 3.52, 95%CI = 1.35–9.24; maximum: OR = 3.43, 95%CI = 1.33–8.82) (Table 3).

### 3.3. Test Results of the Mediation Model

The goodness of fit of the default model was evaluated using a series of fitting coefficients. The χ^2^/df index of the mediation model was 1.726 (1 < χ^2^/df <3, *p* < 0.001). The SRMR value was 0.007. The RMSEA value was 0.014. Both indices were <0.1. The values of the goodness-of-fit index (GFI), incremental fit index (IFI), non-normed fit index (NNFI), and comparative fit index (CFI) were all >0.9, indicating that the mediation model fitted well (Table 4).

### 3.4. Mediation Model of Sleep Duration

**Figure 2:**
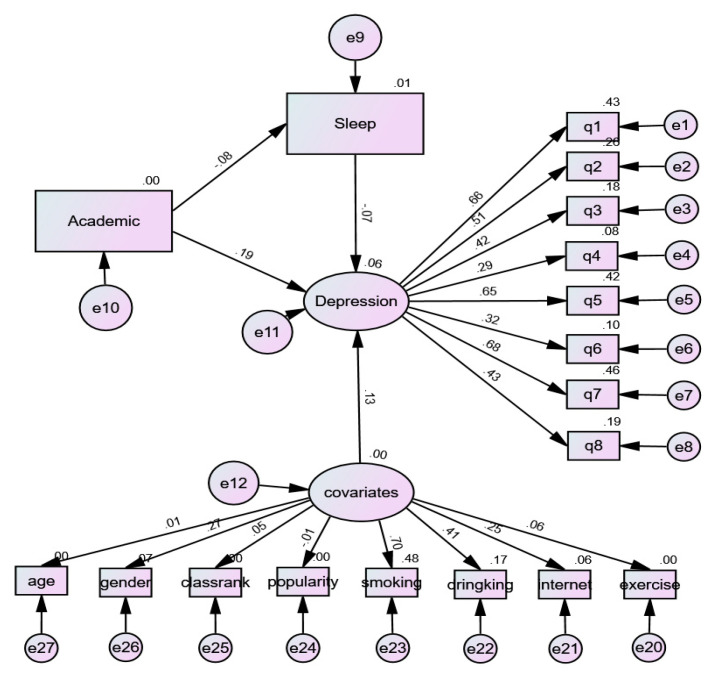
Results of the validated model.

The construction of the structural equation model is shown in Figure 2. According to the regression coefficient of the AMOS structural equation model and the Bootstrap test results, each path display reached a significant level (*p* < 0.05). In model 1, the standardized path coefficient of academic pressure on depression symptoms was 0.197 (*p* < 0.001), which suggested that academic pressure had a significant positive effect on depressive symptoms. By taking sleep duration as the mediating variable, the standardized path coefficient of the effect of academic pressure on sleep duration was −0.084 (*p* < 0.001), which suggested that academic pressure had a significant negative effect on sleep duration. The standardized path coefficient of sleep duration classification on the depressive symptoms was −0.066 (*p* < 0.05), suggesting sleep duration had a significant negative impact on the depressive symptoms. The standardized path coefficient of academic pressure on depressive symptoms was 0.197 (*p* < 0.001), suggesting that academic pressure had a significant positive impact on depressive symptoms. When sleep duration was set as the mediating variable, the standardized path coefficient of academic pressure and depressive symptoms decreased, and the mediating effect value of sleep duration was 0.006 (a * b). Then, the Bootstrap method was used to verify the mediating effect of 95% BootCI (0.001–0.012), and the results suggested that sleep duration was a partial mediator (Table 5).

## 4. Discussion

In this study, we found that approximately 6.49% of the Chinese adolescents had depressive symptoms. Sleep duration of <6 h/night and academic pressure were both associated with an increased risk of depressive symptoms in adolescents. After adjusting for potential factors, the adolescents who reported high/maximum academic pressure remained significantly with a higher risk of depressive symptoms. Meanwhile, the multiplicative interaction between sleep duration and academic pressure was statistically associated with depressive symptoms in adolescents. The mediating effect model showed that sleep duration played a partial mediating role in the relationship between academic pressure and depressive symptoms.

In this study, depressive symptoms among Chinese adolescents were consistent with related studies’ findings, which showed 6.4% of high school students had depressive symptoms [32]. However, the incidence of depressive symptoms was lower than that in children and adolescents reported by an earlier meta-analysis (19.85%) [4]. The differences may be attributed to the sample size, as well as regional and environmental factors. Overall, the prevalence of depression among Chinese adolescents suggests that depressive symptoms are a growing public health problem. In this study, the percentage of students who reported a sleep duration of >8 h/night (54.8%) was consistent with related research findings [32,33]. Sleep duration in different regions and countries was related to the sample’s nature (such as gender and age) and other environmental factors (such as schooling time or cultural background). This study also detected significant differences in gender, age, exercise duration, internet use time (for entertainment), class rank, popularity distribution, smoking, and drinking between adolescents with and without depressive symptoms. These results may help identify adolescents who are more inclined to develop depressive symptoms. The covariate effects of these variables on the associations between sleep duration, academic stress, and depressive symptoms should also be considered.

Our study found that a sleep duration of <6 h/night was associated with an increased risk of depressive symptoms in adolescents. In the mediation model, the standardized path coefficient of the effect of sleep duration classification on the depressive symptoms was −0.066 (*p* < 0.05), indicating that sleep duration had a significant negative impact on the depressive symptoms. Sleep health is essentially correlated with physical and mental health outcomes in adolescents [34], and many studies have found that adolescents whose sleep duration was shorter than the recommended level were more likely to develop emotional problems such as depression, anxiety, and anger [35]. Short sleep duration also increases the risk of depressive symptoms in early teenagers [36,37,38] and is associated with more negative emotions [39]. On the other hand, long sleep duration also is linked to an increased risk of depressive symptoms in adolescents [40]. Studies have found a U-shaped relationship between sleep duration and suicidal ideation among adolescents [41], and a Chinese survey showed that short sleep duration was associated with an increased risk of NSSI among adolescents [42]. Therefore, it is essential to pay attention to and adjust adolescents’ sleep conditions to improve their health status and reduce harmful behaviors. After adjusting for the basic sociodemographic characteristics, adolescents with a sleep duration <6 h/night were still at a higher risk for depressive symptoms.

In other populations, a shorter sleep duration, compared with the standard level, was independently associated with higher levels of depressive symptoms in adults [43]. Women who slept less than 8 h a night had a higher risk of depression [44]. Studies on college students have shown that sleepiness and sleep debt play a mediating role in the relationship between sleep deprivation and the risk of depression and anxiety, and sleep deprivation also increases the risk of depression [45]. In adults, those who sleep for 4 or 5 h a night are more likely to have depressive symptoms than those who sleep for 7 or 8 h a night [46,47], which is consistent with the results of the related studies in middle-aged and elderly people [48]. A Japanese study also found that insomnia was a risk factor for depression among Japanese hospital employees, and the insomnia score could be a valuable marker for early detection of depression [49]. One possible explanation is that people who sleep for an unusual amount of time (short or long) may have greater stress severity than those who sleep for a standard amount of time [46].

Adolescents may use the internet for recreation and participate in physical activity to improve their psychological well-being, and there are studies showing that adolescents who exercise more have lower scores for depressive symptoms [50]. Previous research has found that adolescents who spend more time online for entertainment are more likely to sleep less than the recommended level [35]. Controlling the duration and frequency that teenagers use electronic media has positive effects on their sleep conditions and mood [51]. A Japanese study has found that excessive entertainment time on the internet is more likely to lead to depression [52]. Another Bangladeshi study has found that harmful lifestyles among adolescents (such as lack of physical activity, alcohol consumption, and short sleep duration) are positively associated with depressive symptoms [53]. Relevant studies have also found that some adolescents sacrifice sleep time to obtain extended time on internet entertainment, and consequently, the increase of internet entertainment time will increase the risk of depressive disorder in adolescents to a certain extent [52].

This study also found that academic pressure increased the risk of depressive symptoms in adolescents. The subjects who reported higher academic pressure were significantly associated with a higher risk of depressive disorder both before and after adjusting for potential confounding factors. In the mediation model, the standardized path coefficient of academic pressure on depressive symptoms was 0.191 (*p* < 0.001), which suggested that academic pressure had a significant positive impact on depressive symptoms. The results of a survey on rural Chinese adolescents suggest that high academic pressure can also increase the occurrence of excessive daytime sleepiness and EDS [54]. Academic pressure is a well-known risk factor for depressive symptoms in adolescents with poor health outcomes.

Studies on the mechanism suggest that short sleep duration is associated with low RS-FC in the amygdala and in aspects involved in emotional regulation, such as the ventral anterior cingulate cortex, anterior central gyrus, and superior temporal gyrus. The midpoint of sleep is associated with altered connectivity in several different brain regions, including the superior limbic gyrus and the posterior central gyrus, which are involved in intersensory and sensory processing. Sleep time and sleep midpoint are both associated with the second-day RS-FC in emotion-related neural circuits in the cerebral cortex of children and adolescents and are associated with complex patterns related to emotional health; thus, changes in the cortical brain circuits related to sleep may lead to mood-related problems during adolescence [55].

Moreover, the results of this study suggested that there was a significant interaction between sleep duration and academic pressure. Sleep duration of <6 h/night was associated with a significantly increased risk of depressive symptoms among adolescents with moderate and high academic pressure. The mediating effect model showed that sleep duration played a partial mediating role in the relationship between academic pressure and depressive symptoms. A possible reason is that different levels of pressure can lead to differences in sleep quality and sleep duration among adolescents. Moderate-to-high levels of academic pressure can lead to shorter sleep duration and more inferior sleep quality among adolescents, and therefore, the affected individuals are more likely to develop depressive symptoms. Some other studies have found that higher school stress is associated with less sleep than the recommended level for older teenagers [35]. As students grow older, their bedtime is gradually changing to later hours while the wake-up time for early morning schools stays the same, leading to a decline in the total sleep duration, and consequently, older teenagers are also more prone to depression [56]. Therefore, this study suggests that teachers and parents should timely understand adolescents’ academic pressure and sleep duration, appropriately reduce their learning pressure, pay attention to their daily lifestyle, and help mitigate their depressive symptoms by adopting corresponding preventive measures. In addition, at the social level, schools and education departments should also properly reduce students’ academic pressure, reduce extracurricular homework, and other factors that might affect students’ sleep duration. Society should pay attention to adolescents’ mental health status and improve the mental health level of adolescents in China.

The results of this study provide a theoretical basis for the risk factors related to depression. The strength of this study lies in the use of national survey data. CFPS is a large nationwide social tracking project with comprehensive coverage and strong representativeness, and its research results are highly representative. Nevertheless, there are some limitations to this study, namely, (1) this is a cross-sectional survey, and thus, no causal association can be drawn; (2) all questions were self-reported; therefore, the results might be biased. The sleep variable only included the sleep duration, while detailed information such as sleep quality was not asked. In addition, self-reported academic pressure might also be biased; (3) the disease history of adolescents, especially psychosocial disorders, was not included in this study, which might influence the results; (4) adolescents aged 10 years old as defined by the WHO were not included in this study; (5) some potentially unknown confounders might not have been taken into account. Therefore, future studies should pay more attention to scientific measurement methods for variables such as sleep duration and academic pressure. Moreover, studies also should use prospective studies with larger sample sizes to explore causal associations and adopt more rigorous scientific research design methods in the future.

## 5. Conclusions

In conclusion, this study demonstrated that a sleep duration of <6 h/night and higher academic pressure were both associated with an increased risk of depressive symptoms among Chinese adolescents. The multiplicative interaction between sleep duration and learning stress was statistically related to depressive symptoms in adolescents. After performing stratified analysis, the results indicated that adolescents with moderate and high academic pressure who slept for <6 h/night had a greater risk of developing depressive symptoms. The association between academic pressure and depressive symptoms was mediated by sleep duration, and sleep duration is a partial mediator. Therefore, it is essential to mitigate the academic pressure of adolescents to increase their sleep duration and further reduce the occurrence of depressive symptoms by adopting corresponding preventive measures.

## Figures and Tables

**Table 1:** Baseline characteristics among 3724 adolescents.

Variable	*n*	CES-D Scores ^&^	*p*-Value ^#^	Depressive Symptoms Yes (*n* = 242)%	No (*n* = 3482)%	*p*-Value ^#^
**Age(years) ^&^**	14.9(2.6)	-	-	15.4(2.6)	14.9(2.6)	0.007 *
Gender						
Girls	1789	4.4(3.2)	0.005 *	137(7.7)	1652(92.3)	0.006 *
Boys	1935	4.1(3.0)		105(5.4)	1830(94.6)	
**Household socioeconomic status**						
Low	1287	4.3(3.2)	0.614	87(6.8)	1200(93.2)	0.816
Medium	2029	4.3(3.1)		131(6.5)	1898(93.5)	
High	408	4.2(3.0)		24(5.9)	384(94.1)	
**Exercise duration/day, min**						
>30	1915	4.3(3.1)	0.091	113(5.9)	1802(94.1)	0.041 *
30–60	1147	4.3(3.3)		92(8.0)	1055(92.0)	
>60	662	4.0(2.9)		37(5.6)	625(94.4)	
**Internet use time (for** **entertainment)/day, h ^&^**	1.7(2.0)	-	-	2.2(2.3)	1.7(2.0)	<0.001 **
**Sleep duration/night, h**						
<6	107	5.5(3.7)	<0.001 **	16(15.0)	91(85.0)	0.001 **
6–8	1577	4.5(3.1)		105(6.7)	1472(93.3)	
>8	2040	4.1(3.1)		121(5.9)	1919(94.1)	
**Midday napping**						
Yes	1596	4.4(3.1)	0.131	101(6.3)	1495(93.7)	0.715
No	2128	4.2(3.2)		141(6.6)	1987(93.4)	
**Smoking**						
Yes	175	5.5(3.2)	<0.001 **	22(12.6)	153(87.4)	0.001 **
No	3549	4.2(3.1)		220(6.2)	3329(93.8)	
**Drinking**						
Yes	88	6.3(3.2)	<0.001 **	14(15.9)	74(84.1)	<0.001 **
No	3383	4.2(3.1)		228(6.3)	3408(93.7)	
**Class rank**						
0–10%	946	3.9(2.9)	<0.001 **	40(4.2)	905(95.8)	0.008 *
11–25%	1001	4.2(3.1)		65(6.5)	936(93.5)	
25–50%	1119	4.4(3.1)		80(7.1)	1039(92.9)	
51–75%	426	4.5(3.5)		37(8.7)	389(91.3)	
76–100%	232	5.1(3.1)		20(8.6)	212(91.4)	
**Academic pressure**						
minimum	394	3.4(3.0)	<0.001 **	13(3.3)	381(96.3)	<0.001 **
low	678	3.8(3.1)		40(5.9)	638(94.1)	
moderate	1622	4.2(3.0)		93(5.7)	1529(94.3)	
high	786	4.8(3.1)		64(8.1)	722(91.1)	
maximum	244	5.4(3.6)		32(13.1)	212(86.9)	
**Popularity relation**						
poor	143	5.8(3.8)	<0.001 **	27(18.9)	116(81.1)	<0.001 **
average	1844	4.6(3.1)		126(6.8)	1718(93.2)	
good	1737	3.8(3.0)		89(5.1)	1648(94.9)	
**BMI z-score**						
underweight	428	4.2(2.8)	0.302	25(5.8)	403(94.2)	0.848
Normal weight	2733	4.3(3.1)		181(6.6)	2552(93.4)	
overweight	417	4.0(3.1)		25(6.0)	392(94.0)	
obesity	146	4.3(3.4)		11(7.5)	135(92.5)	

Abbreviations: BMI, body mass index; “^&^” data are presented as the means (standard deviation: S.D.); “^#^” the t-tests and one-way ANOVA were used for continuous variables; chi-square tests were used for categorical variables. * *p* < 0.05, ** *p* < 0.001.

**Table 2:** Respective association of sleep duration, academic pressure, and their interaction item with depressive symptoms.

Variable	Depressive Symptoms Model A OR (95% CI)	Model B aOR (95% CI)	Model C aOR (95% CI)	Model D aOR (95% CI)
**Sleep duration (h/night)**				
<6	2.35(1.32–4.17) *	2.39(1.33–4.32) *	2.53(1.41–4.58) *	-
6–8	1.00(reference)	1.00(reference)	1.00(reference)	-
>8	0.91(0.69–1.19)	0.88(0.67–1.17)	0.91(0.69–1.19)	-
**Academic pressure**				
minimum	0.56(0.31–1.02)	0.59(0.32–1.07)	-	0.60(0.33–1.09)
low	1.04(0.71–1.53)	1.09(0.74–1.60)	-	1.13(0.77–1.67)
moderate	1.00(reference)	1.00(reference)	-	1.00(reference)
high	1.43(1.02–1.99) *	1.43(1.02–2.00) *	-	1.44(1.03–2.02) *
maximum	2.43(1.58–3.73) **	2.54(1.64–3.93) **	-	2.45(1.58–3.81) **
**Interaction item (Sleep duration ×Academic pressure) ^#^**				
multiplicative interaction (*p*)	0.001 **	0.001 **	-	-

Abbreviations: OR, odds ratio; aOR, adjusted odds ratio; CI, confidence interval; “-” no data available. Model A was the unadjusted model. Model B adjusted for age, gender, exercise duration, internet use time (for entertainment), class rank, smoking, drinking, popularity relation. Model C adjusted for age, gender, exercise duration, internet use time (for entertainment), class rank, smoking, drinking, popularity relation, and academic pressure. Model D adjusted for age, gender, exercise duration, internet use time (for entertainment), class rank, smoking, drinking, popularity relation, and sleep duration. ^#^ Sleep duration, academic pressure, and the interaction item between sleep duration and academic pressure were entered simultaneously into the multivariable logistic regression models. * *p* < 0.05, ** *p* < 0.001.

**Table 3:** Association between sleep duration and depressive symptoms stratified by academic pressure.

Variable	Academic Pressure Minimum aOR ^e^ (95% CI)	Low aOR ^e^ (95% CI)	Moderate aOR ^e^ (95% CI)	High aOR ^e^ (95% CI)	Maximum aOR ^e^ (95% CI)
**Sleep duration** **(h/night)**					
<6	-	0.74(0.07–7.3)	3.52(1.35–9.24) *	3.43(1.33–8.82) *	0.73(0.08–6.67)
6–8	1.00(reference)	1.00(reference)	1.00(reference)	1.00(reference)	1.00(reference)
>8	2.81(0.60–13.18)	0.76(0.38–1.51)	0.83(0.53–1.28)	0.72(0.41–1.26)	1.32(0.57–3.06)

Abbreviations: aOR, adjusted odds ratio; CI, confidence interval; “-” no data available; “^e^” was adjusted for age, gender, exercise duration, internet use time (for entertainment), class rank, smoking, drinking, popularity relations. * *p* < 0.05.

**Table 4:** Fitting indices for default model.

χ^2^	df	χ^2^/df	RMSER	SRMR	NNFI	GFI	IFI	CFI	NFI	NNFI	*p*
13.810	8.000	1.726	0.014	0.007	0.967	0.994	0.994	0.994	0.986	0.957	0.087

**Table 5:** Regression coefficients and the bootstrap test of the mediation model.

Model	Path	Standardized Estimate	Estimate	S.E.	C.R.	Bias-Corrected Percentile	*p*
						Lower Upper	
**Model 1 ^a^**							
	AP→DS(c)	0.197	0.085	0.008	10.485	0.158 0.233	<0.001 **
**Model 2 ^b^**							
	AP→SD(a)	−0.084	−0.045	0.009	−5.159	−0.116 −0.051	<0.001 **
	SD→DS(b)	−0.066	−0.053	0.015	−3.557	−0.103 −0.028	<0.001 **
**Model 3 ^c^**							
	AP→DS(c’)	0.191	0.082	0.008	10.187	0.152 0.227	<0.001 **

Adjusting for age, gender, exercise duration, internet use time (for entertainment), class rank, smoking, drinking, popularity relations. Abbreviations: A.P., academic pressure; D.S., depressive symptom; SD, sleep duration; S.E stander error; C.R. composite reliability; “^a^”, model 1; “^b^”, model 2; “^c^”, model 3. ** *p* < 0.001.

## Data Availability

Original data in this study are obtained from the Institute of Social Science Survey of Peking University and are available at https://www.isss.pku.edu.cn/cfps/index.htm (registration and approval needed, accessed on 5 June 2021).
